# Cerebellar ataxia, neuropathy and vestibular areflexia syndrome (CANVAS)

**DOI:** 10.1055/s-0045-1813234

**Published:** 2025-12-02

**Authors:** Adolfo M. Bronstein

**Affiliations:** 1 Imperial College London, London, United Kingdom.

In this issue Fernandes et al from Porto, Portugal, present their findings in a group of 15 patients with Cerebellar Atrophy, Neuropathy, Vestibular Areflexia syndrome, or CANVAS for short. 1 This condition exemplifies the pace of research-driven progress in medical science: The first two patients were reported in London in 1990 2 and by 1998 patients with this combination of findings made 13% (7/53) in a series of patients with bilateral vestibular failure reported by the same group. 3 In 2011 Szmulewicz et al 4 in Sydney fully characterised the syndrome, contributed to the pathology and, very importantly, created the catchy acronym that made CANVAS a recognizable disorder first in neuro-otology, then in peripheral neurology and cerebellar subspecialty circles. The gene, a biallelic expansion of an intronic repeat in RFC1 was found by Cortese et al 5 in London in 2019 and this progression of discoveries has placed CANVAS into the realms of general neurology. In this wider context, the paper by Fernandes et al is very welcome as it effectively reviews the topic in a general neuro-psychiatry journal in the Portuguese language world. Most specialists agree that CANVAS still is under-recognised and under-diagnosed and, therefore, this paper will go some way into remedying this.

Fernandes et al report their clinical and laboratory findings in 15 patients with a positive gene test. This is important because, as Portuguese speaking neurologists know, certain neuro-genetic conditions such as SCA 3 (Machado-Joseph disease) are more frequent amongst their patients than in other international communities. 6 Although there are no consistent statistics comparing the clinical or genetic impact of CANVAS/RFC1 disease internationally, it is clear that the condition is here to stay amongst descendants of Portuguese origin.

An important finding is that in this group of genetically segregated patients the full triad of symptoms is only present in approximately 2/3 of patients, supporting the notion that clinicians must consider RFC1 as a possible diagnosis for their patients with just two or even one of the components of the syndrome.

For those readers not very familiar with CANVAS or specialised in vestibular or oculo-motor matters, I am tempted to say that in most patients the diagnosis is easy. A quick recapitulation is needed though. Slow phase eye movements are generated by two separate physiological systems: the vestibulo-ocular reflex (VOR), induced by head movements, and the cerebellar-driven smooth pursuit system, generated by following a slowly moving visual target. The critical concept is that CANVAS obliterates both systems and, therefore, most patients with the condition cannot generate normal slow-phase eye movements. 2 Hence, asking your patient to ***slowly*** oscillate the head from side to side and up-down while they fixate on your nose (the doll's eye manoeuvre) will reveal a very broken-up or “saccadic” eye movement. This is an easy way to recognise this oculo-motor finding, highly specific to these patients because of the combined vestibular and cerebellar damage 4 –a combination that apart from CANVAS not many other disorders do. Add a polyneuropathy and other cerebellar oculo-motor findings like downbeat nystagmus and the diagnosis is made. Although the paper by Fernandes et al show that ancillary testing increases the diagnostic sensitivity this is not very impressive. In their own words: “From a clinical standpoint, the classical triad of RFC1-related disorder symptoms was evident in only 10 patients (66.6%). However, this number increased to 11 (73.3%) upon comprehensive neurological examination and to 12 (80.0%) when including ancillary exams.”

All in all, this paper is a good addition to the Arquivos de Neuro-Psiquiatria that most readers will surely enjoy.
